# Total Phenolic, Anthocyanin, Catechins, DPPH Radical Scavenging Activity, and Toxicity of* Lepisanthes alata (Blume) Leenh*

**DOI:** 10.1155/2019/9703176

**Published:** 2019-06-02

**Authors:** Tuty Anggraini, Syafni Wilma, Daimon Syukri, Fauzan Azima

**Affiliations:** Faculty of Agricultural Technology, Andalas University, Padang 25163, Indonesia

## Abstract

Anthocyanins and catechin are natural antioxidants presented in many plants*. Lepisanthes alata (Blume) Leenh* is a plant with fruit that ripens to an intense red. This coloring suggests a high polyphenol content. However, limited information is available regarding the polyphenol or other antioxidant content in this fruit or its suitability as a food additive. The purpose of this research was to determine the total phenolic, total monomeric anthocyanin, catechin, epicatechin and epigallocatechin gallate content, DPPH radical scavenging activity, and toxicity in rind, flesh, seed, and whole fruit of* L. alata*. This research was conducted using an exploratory method with four analyses; samples from six parts of the plant were analyzed for polyphenols (rind, fruit pulp, seeds, whole fruit, bark, and leaves), four for anthocyanins (rind, fruit pulp, seeds, and whole fruit), and six parts of the plant for DPPH radical scavenging activity in water, methanol, and ethanol (rind, fruit pulp, seeds, whole fruit, bark, and leaves) and toxicity. This plant was found to contain high levels of polyphenol; the lowest level was measured in the flesh (0.64 mg GAE/g of DW) and the highest in the whole fruit (2.87 mg GAE/g of DW). The lowest anthocyanin content is found in the flesh and the highest in the rind with the respective average values of 672.27 mg/100 g FW and 1462.82 mg/100 g FW. Epicatechin is the major catechin in whole fruit and bark of* L. alata*.* L. alata* DPPH radical scavenging activity was in the range of 21.23 to 92.5% depending on the solvent, and the highest activity was recorded in bark in ethanol extract. No toxins were found in any part of the plant indicating that an extract from it could be safely used as a natural antioxidant supplement in processed foods to protect against free radicals.

## 1. Introduction

Free radicals are highly reactive compounds that can produce oxidative stress on the body contributing to diseases such as atherosclerosis or cancer. To achieve atomic stability, free radicals oxidize surrounding molecules to obtain a pair of electrons. This oxidative damage can be counteracted with primary or secondary antioxidants [1–7]. The antioxidant activity of bioactive compounds has been found to have the ability to maintain cell structure and functions efficiently and inhibit free radicals and lipid peroxidation reactions and prevent other oxidative damage [8–11].

Anthocyanins are a class of naturally occurring antioxidants that belongs to the group of polyphenolic compounds. They are known as pigments abundant in the highly colored plants and impart red, blue, purple, or black color. For instance, both black and red rice contain high levels of anthocyanins [12–14]. As color is a major component in the attractiveness of food or beverage for consumers, anthocyanin with their lack of toxicity and antioxidative properties are in growing demand as healthier alternatives to artificial food colorants. Blueberry with its high polyphenol content, including purple anthocyanin pigments, is commonly used as a colorant in muffins, salad, syrup, bread, and fruit juices [15]. Some of these naturally occurring pigments have proven health benefits. For example, the purple-coloring mulberry anthocyanin extract has been shown to potentially protect hepatocytes against oxidative stress during hyperglycemia in HepG2 cells [16].

*Lepisanthes alata (Blume) Leenh* (*L. alata*) belongs to the Sapindaceae family also known as Belimbing Cina or Malay Cherry. It grows wild in the forest in Java, Sumatra, and Malaysia and is also used as an ornamental tree and to provide shade. It has already been established that the leaves of* L. alata* contain polyphenols: proanthocyanidin, epicatechins, and epigallocatechins [17]. The plants also have dark purple fruit suggesting high levels of anthocyanin. It would seem likely, therefore, that parts of this plant may have value as a component of functional foods.

However, there is still a lack of detailed information about the nature of the antioxidants in various parts of* L. alata*. Therefore, this study aims to identify potential antioxidants in the fruit, leaves, and bark of this plant. DPPH radical scavenging activity was determined, and the contents of polyphenols, anthocyanins, catechin, epicatechin, and epigallocatechin gallate were analysed. To check the safety for use as food additives, a toxicity test of these parts of the plant was conducted.

## 2. Material and Methods

### 2.1. Chemicals

1,1-Diphenyl-2-picrylhydrazyl (DPPH), ethanol, methanol, buffer potassium chloride, sodium acetate buffer, HCl, Folin Ciocalteu, Na_2_CO_3_, Gallic acid, sodium hydroxide, DMSO, phosphoric acid, tetrahydrofuran, acetonitrile, catechin, epicatechin, and epigallocatechin gallate standard are obtained from Sigma Aldrich Company, Germany.

### 2.2. Plant Material

Whole mature fruit, young leaves, and bark of* L. alata* from the area of Belimbing, Padang, Indonesia, were used.

### 2.3. Total Phenolic Content

The total phenolic content of the samples was determined using the Folin Ciocalteu colorimetric method as described by Tumbarski et al. (2019) [18]. One g samples (of rind, flesh, seeds, whole fruit, leaves, and bark) were put in 10 mL of methanol and then put into a homogenizer for 15 minutes. One mL of the resulting crude extract was added to 2 mL of distilled water and 1 mL Folin Ciocalteu reagent and homogenized for 5 minutes. One mL of 5% Na_2_CO_3_ was added and the mixture incubated 2 h at room temperature. The absorbance was then measured at 725 nm. Samples were calculated using gallic acid (3,4,5-trihydroxy benzoic acid (C_6_H_2_ (OH_3_) CO_2_H)) as the standard and total phenolic content was expressed as mg gallic acid equivalents (GAE)/100 g dry weight (DW).

### 2.4. Total Monomeric Anthocyanin Content

The total monomeric anthocyanin content was determined as described by Coklar and Akbulut (2017) [19] using the differential method. Each sample (rind, pulp, seeds, and the whole fruit) was homogenized and then 1 gram was added to about 9 mL of methanol and 1 mL of 27% HCl. A 0.5 ml sample of crude extract was poured into two reaction tubes. KCl buffer (0.025 M) pH 1 was added to the first, and 9.5 mL sodium acetate buffer (0.4 M) pH 4.5 was added to the second. After 15 minutes, the absorbance of the sample was measured using a spectrophotometer at 515 nm and 700 nm. The anthocyanin content (cyanidin-3-glucoside equivalents, mg/100g) was calculated as follows:

(1) $$\begin{array}{c} \text{Anthocyanin content}=\frac{A\times MW\times DF\times 100}{\varepsilon \times 1} \end{array}$$

where A = pH 1.0 (A 520 – A 700 nm) – pH 4.5 ((A 520 – A 700 nm); MW = molecular weight of cyanidin-3-glucoside (449.2 g/mol); DF = dilution factor; and *ε* = molar extinction coefficient of cyanidin-3-glucoside (26.900 L/mol x cm).

### 2.5. Catechin, Epicatechin, and Epigallocatechin Gallate [20]

Catechins were determined using a HPLC series system with a high-pressure gradient pump, an autosampler, a column oven (Prominence HPLC 20A Shimadzu), and a reverse-phase chromatographic column (Agilent Zorbax SB-C18 column) coupled to a UV visible detector. The eluents were (A) water with 0.2% of phosphoric acid and 1% of tetrahydrofuran and (B) acetonitrile with 1% of tetrahydrofuran. The separation was developed under step gradient polarity system that was from 5 to 25% B for 20 min at a flow rate of 0.8 ml/min where the detection wavelength was at 230 nm.

### 2.6. DPPH Assay [19]

One-gram samples (rind, flesh, seeds, whole fruit, leave, and bark) were added to 10 mL of either water, methanol, or ethanol then homogenized. The crude extract samples were mixed with 3.9 ml of methanol and 1 ml of a DPPH solution (1mM in methanol) in a test-tube and the absorbance was measured at 517 nm after 30 minutes of incubation.

DPPH radical scavenging activity was calculated as follows:

(2) DPPH  radical  scavenging  activity=1−(A517sampleA517blank  x  100 %

### 2.7. Brine Shrimp Lethality Test (BSLT)

This was conducted as described by (Supraja et al., 2018) [21]. Ten shrimps were placed in a vial containing 5 ml of seawater and a 1000, 100, or 10 ppm sample extract. A control with no extract was also used. After 24 hours, the number of dead shrimps was observed. This was done with three replications for each concentration.

### 2.8. Statistical Analysis

The results of all experiments were expressed as mean ± SD of triplicate measurements.

## 3. Results and Discussion

**Figure 1:**
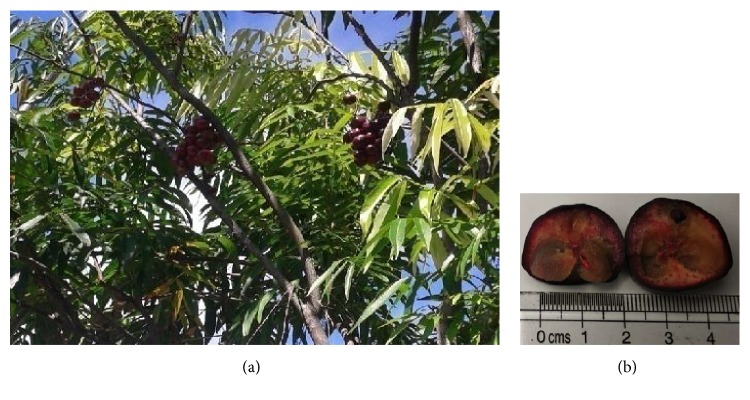
*L. alata* plant (a).* L. alata* fruit (b).

The* L. alata* plant can be seen in Figure 1.

### 3.1. Total Phenolic Content

**Figure 2:**
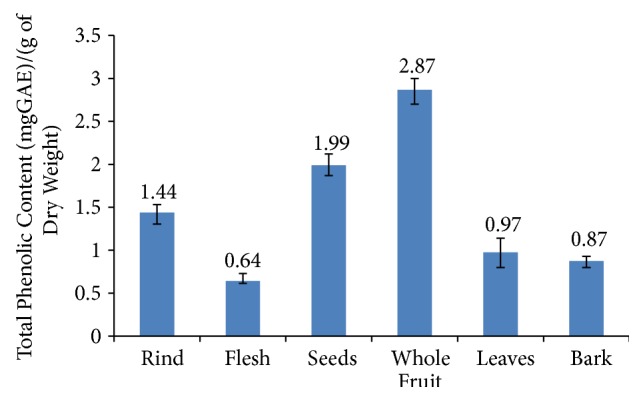
Total phenolic content of* L. alata*.

Polyphenols can take a number of different chemical forms. Anthocyanins are common in fruit, while ellagitannins have been found to be the major phenol compounds in other parts of plants including many leaves [22]. Several studies have reported that phenolic compounds are responsible for the DPPH radical scavenging activity as evidenced by the positive correlation between DPPH activity and phenolic content [23–25]. The total phenolic content was measured for the rind, flesh, seeds, whole fruit, leaves, and bark of* L. alata* using methanol extract. The test results can be seen in Figure 2.

The highest polyphenol content was found in the whole fruit, followed by the seeds and then the rind and leaves. The bark and flesh had the lowest polyphenol content. The total phenolic content ranged from 0.64 to 2.87 mg GAE /g of DW. Surprisingly, whole fruit extract showed a higher phenolic content compared to any individual part of the fruit despite the flesh, which makes up the largest mass of the fruit, having a low phenolic content. This may be due to some synergic reaction increasing the release of the phenolic compounds in the whole fruit. Such complex interactions have been reported in other polyphenol-containing plants and plant products. Research conducted by Lopez et al. (2019) [26] found that a synergic reaction with fructosylation improved the bioactivity of phenolic compounds in* Gluconacetobacter diazotrophicus*. Exposure to air induces the formation of hydroxyl radicals in wine which has been found to enhance the phenolic (caffeic acid, protocatechuic, p coumaric acid, and gentisic and syringic acid) content [27]. Apart from the value of the whole fruit, the total polyphenols of different parts of the fruit showed a similar pattern to the total monomeric anthocyanin. The phenolic contents of the seeds and fruit are relatively high. Although absolute values cannot be directly compared, it would seem that* L. alata* is a rich plant source of polyphenols. However, an ethanol extract of* Nitraria tangutorum* seed meal has been found to have 9.47 GAE mg/g polyphenols [28].

### 3.2. Total Monomeric Anthocyanin Content

Anthocyanins are one class of phenolic compounds and are natural flavonoid pigments that have aroma and colors from red to deep purple [29]. The particular anthocyanin compounds in a plant depend on the cultivar and species [30, 31].

**Figure 3:**
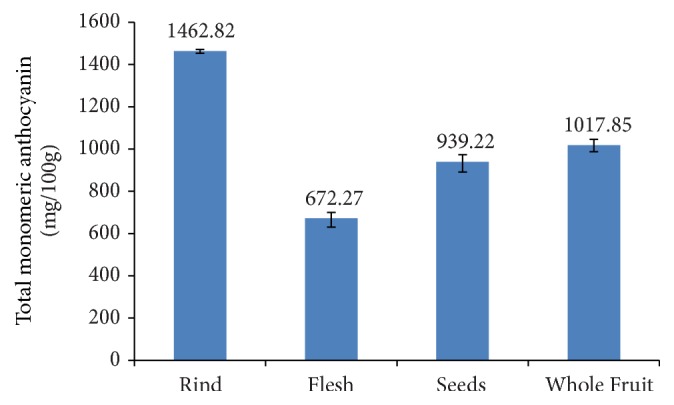
Total anthocyanin of* L. alata* fruit.

Monomeric anthocyanin content of the fruit (rind, flesh, seeds, and whole fruit) was tested because its purple color indicates the presence of these compounds. As other parts of the plant did not show these colorings, they were not tested. The coloring of* Syzygium cumini* fruit is similar to that of* L. alata* and is due to anthocyanin pigments cyanidin 3-glucoside and malvidin 3-glucoside [32]. Purple blueberry cultivars contain many different anthocyanins: delphinidin, cyanidin, petunidin, peonidin, and malvidin glycoside [33]. The dark purple* Lonicera caerulea* berry contains anthocyanin cyaniding-3-acetylhexoside and peonidin-3-acetylhexoside [34]. The purple-colored pomace of black carrots contains two cyanidins: cyanidin-3-xyloside-galactoside-glucoside-ferulic acid and cyanidin-3-xyloside-galactoside [35]. The anthocyanin content of rind, flesh, seed, and whole fruit can be seen in Figure 3.

The highest anthocyanin content was found in the rind (1462.82 mg/100 g FW), followed by the whole fruit, and then successively seeds and flesh of* L. alata*. Generally, the more intense the color of the material, the higher the anthocyanin content. Therefore, it is not surprising that the dark red rind should have a higher anthocyanin content than the paler red flesh or the white seeds. Apart from the value of the whole fruit, the total monomeric anthocyanin of different parts of the fruit showed a similar pattern to the total polyphenols.

These results are higher than previously reported results of other purple-colored fruit suggesting that* L. alata* is a particularly rich source of these compounds. Coklar and Akbulut (2017) [19] determined the total anthocyanin content of* Mahonia aquifolium* to be 253.40 mg/100 g FW. The purple bracts of banana (*Musa X paradisiacal*) have been found to contain 32 mg anthocyanin/100 g bracts [36]. The total anthocyanin content in bokbunja was only 3.76 mg/g FW [37].

Further study is needed to identify the particular anthocyanin present in* L. alata* and their potential health benefits.

### 3.3. Catechin, Epicatechin, and Epigallocatechin Gallate

Different result showed catechin, epicatechin, and epigallocatechin content in whole fruit, leaves, and bark. The content of catechin, epicatechin, and epigallocatechin can be seen in Table 1. This study showed for the first time the catechin, epicatechin, and epigallocatechin gallate of fruit, leaves, and bark of* L. alata*.

The bark of* L. alata* had the highest content of epicatechin. Research conducted by Gottumukkala et al. (2014) [38] also found that epicatechin in cocoa is higher than catechin.

### 3.4. DPPH Assay

DPPH radical scavenging activity was measured for the rind, flesh, seeds, whole fruit, plant leaves, and bark of the plants by using three different solvents, organic and polar: aqueous, methanol, and ethanol. These solvents extract different compounds from the plant material according to their polarity [19]. Results can be seen in Table 2.

Table 2 shows the results of DPPH radical scavenging activity of* L. alata* when extracted using different solvents. The DPPH radical scavenging activity differs considerably according to solvent and part of the plant used ranging from 21.23 to 92.52%. For the flesh, the water extract had the highest DPPH radical scavenging activity. The methanol and ethanol extracts of the rind, leaves, seeds, and bark showed higher activity than the water extracts. A similar result was found in the leaves of* Coffea arabica* L. where ethanol extracts showed the higher antioxidant activities than water extracts for five pigments: pheophytin *α*, zeaxanthin, crocetin, *β*-carotene, and chlorophyll b form [39]. A higher DPPH activity was found in the methanol and ethanol extracts of brown seaweed* Sargassum serratifolium* than in the aqueous extract [40]. For black currant fruit, the highest percentage extraction of anthocyanin was found using ethanol as the solvent suggesting that, even for fruit, the antioxidant activity may sometimes be higher with alcohol extracts than with water [41]. However, aqueous extracts of fruit are more likely to be food-safe than ethanol or methanol extracts.

The highest DPPH radical scavenging activity was found in bark followed by seeds, rind, leaves, whole fruit, and flesh. As with the total polyphenol content, the water extract of whole fruit showed higher DPPH radical scavenging activity than that of the constituent parts (the rind, flesh, and seed) indicating that the antioxidant property in one part of the fruit was enhanced by the presence of phenolic or other compounds in another. It would be interesting to investigate if this finding was due to the same apparent synergetic mechanisms as the polyphenol activity. Comparative strength of DPPH radical scavenging activity in other parts of the plant shows some similarity to the total phenolic content: however, while bark had less than half the total phenolic content than seeds or rind, its DPPH radical scavenging activity was similar, possibly indicating the presence of other antioxidant components which could be further investigated.

### 3.5. Brine Shrimp Lethality Test

The toxicity tests using Brine Shrimp Lethality Test (BSLT) on the whole fruit, leaves, and bark of plants can be seen in Table 3.

Brine Shrimp Lethality Test (BSLT) uses a bioassay with larva* Artemia salina* Leach to determine toxicity over a 24-hour period after the administration of the test dose. Table 2 shows the value of LC_50_ (dose that is lethal for half the shrimps) as determined by probit analysis of the response of the shrimp to extracts of* S. alata*. The lowest LC_50_ was 1600 mg/mL for the whole fruit extract indicating that none of the tested parts of* L. alata* are toxic because the LC_50_ value obtained was still > 1000 mg/mL indicating it is safe for consumption.

## 4. Conclusions

*Lepisanthes alata (Blume) Leenh* fruit contains anthocyanin, high total polyphenol, and epicatechin was the major catechin in bark of* L. alata*. Toxicity testing suggested that the parts of the plant tested are safe for consumption. This fact, along with the demonstrated antioxidant properties of these parts of the plant, indicates that it could become a healthy source of additives to enhance color or antioxidant value to food. More research is required to determine the detailed chemical composition of the antioxidant compounds of different parts of the plant and the solvents which will produce the most advantageous extracts for food.

## Figures and Tables

**Table 1:** Catechin, epicatechin, and epigallocatechin gallate of *L. alata*.

Extracts	Catechin (*µ*g/mL)	Epicatechin (*µ*g/mL)	Epigallocatechin gallate(*µ*g/mL)
Whole Fruit	4.70 ± 0.4	161.10 ± 4.1	32.60 ± 2.9
Leaves	10.60 ± 1.1	15.60 ± 3.3	27.95 ± 2.8
Bark	87.60 ± 2.5	355.45 ± 4.8	27.98 ± 2.8

**Table 2:** DPPH radical scavenging activity of *L. alata*.

Extracts	DPPH radical scavenging activity (%)		
	Water	Methanol	Ethanol
Rind	61.61±1.29	86.17±4.67	85.81±0.74
Flesh	47.93±2.33	27.47±1.96	21.23±2.50
Seeds	48.66±2.55	89.58±2.15	90.12±2.19
Whole fruit	69.30±0.91	78.34±1.89	46.20±0.95
Leaves	59.35±2.82	61.71±1.16	79.61±2.48
Bark	49.91±4.23	87.03±4.11	92.52±0.77

**Table 3:** Brine shrimp lethality test of *L. alata*.

Sample	Concentration (ppm)	Average % of mortality	Probit value (Y)	log const. (X)	LC_50_
Whole fruit	10	13.3333	3.8893	1	1597.95
	100	23.3333	4.272	2	
	1000	46.6667	4.9417	3	

Leaves	10	10.0000	3.718	1	3583.44
	100	13.3333	3.8893	2	
	1000	43.3333	4.8233	3	

Bark	10	3.3333	3.2276	1	5360.43
	100	10.0000	3.718	2	
	1000	33.3333	4.5723	3	

## Data Availability

The data used to support the findings of this study are included within the article.
